# An association between *hOGG1* Ser326Cys polymorphism and the risk of bladder cancer in non-smokers: a meta-analysis

**DOI:** 10.1186/1471-2407-12-335

**Published:** 2012-08-02

**Authors:** Changwei Ji, Zhao Liu, Huimei Chen, Hongqian Guo, Changjian Liu

**Affiliations:** 1 Department of Urology, the Affiliated Drum Tower Hospital of Nanjing University Medical School, Nanjing, 210008, China; 2 Department of Vascular Surgery, the Affiliated Drum Tower Hospital of Nanjing University Medical School, Nanjing, 210008, China; 3 Department of Medical Genetics, Nanjing University Medical School, Nanjing, 210093, China

## Abstract

**Background:**

Bladder cancer results from complex interactions between many genetic and environment factors. The polymorphism Ser326Cys in *hOGG1* gene has been reported to be associated with bladder cancer in some studies, though the results remain inconclusive. To explore this relationship of *hOGG1* polymorphism and the susceptibility for bladder cancer and the impact of smoking exposures, a cumulative meta-analysis was performed in this study.

**Methods:**

We extracted the data from the Pubmed database up to January 9, 2012 using the search phrases “*hOGG1*, Ser326Cys polymorphism and bladder cancer”. Seven case–control studies were identified, including 2474 patients and 2408 controls. Four of them provided the analysis of smoking effects, with 1372 smokers and 947 non-smokers. The odds ratios (ORs) and associated 95 % confidence intervals (CIs) were calculated using fixed- or random- effects models.

**Results:**

Regarding the overall association between the *hOGG1* 326Cys allele and bladder cancer risk, the meta-analysis did not reveal a significant effect in the additive model (OR: 1.06, 95 % CI: 0.96-1.26; *p* = 0.49), the recessive genetic model (OR: 1.05, 95 % CI: 0.65-1.70; *p* = 0.85) or the dominant genetic model (OR: 1.07, 95 % CI: 0.87-1.32; *p* = 0.53). Similarly, no significant relationship was observed in the stratified analysis by ethnicity, study design and Hardy-Weinberg equilibrium (all *p* > 0.05). In the non-smokers, however, *hOGG1* 326Cys allele significantly increased the risk for bladder cancer and the ORs in the additive model, homozygote contrast and recessive genetic model were 1.59 (*p* = 0.02), 2.53(*p* = 0.003) and 2.41(*p* = 0.0005), respectively. Nevertheless, in the smoker subgroup, similar findings could not be found in all genetic models (all *p* > 0.05).

**Conclusions:**

The association between the *hOGG1* 326Cys allele and bladder cancer was significant in non-smoker population, while was non-detectable in common or smoker populations. This meta-analysis suggests that the *hOGG1* Ser326Cys polymorphism may be a risk factor for bladder cancer without exposure to smoking. Further functional studies are needed to elucidate the gene polymorphism-bladder cancer relationship and gene-environment interactions.

## Background

Bladder cancer remains one of the most common malignant diseases around the world [1]. The occurrence of bladder cancer resulted from many exogenous and endogenous factors, such as cigarette smoking, genetic background, and capability to repair damaged DNAs [2-7]. Indeed, the DNA repairing systems, composed of many DNA repair genes, play a critical role in removing damaged genes, maintaining the genomic integrity and preventing carcinogenesis. The human 8-oxoguanine glycosylase gene *(hOGG1*) is such a commonly-studied DNA repair gene [8,9]. It encodes the 8-oxoguanine DNA glycosylase, a key enzyme in the removal of 8-oxodeoxyguanosines generated by oxidative stress and highly mutagenic. The polymorphisms in the *hOGG1* genes may impair their abilities to repair damaged genes, leading to genetic instability and carcinogenesis [10].

Among the many polymorphisms of *hOGG1* genes, the Ser326Cys polymorphism has received much attention in recent years. It is an amino acid substitution of serine (Ser) with a cysteine (Cys) in exon 7 of *hOGG1* gene [11,12], and the 326Cys allele reduces DNA repair activity and increases the cancer risk [13-16]. In pursuit of identifying an association between the Ser326Cys polymorphism and bladder cancer risk, many studies have been conducted. However, the results were inconsistent and even contradictory [17-23], presumably due to the relatively small samples of individual studies, various genetic background and possible selective bias.

Herein, we performed a meta-analysis on the previously reported studies, and investigated whether the Ser326Cys polymorphism increases the bladder cancer risk. Specifically, a stratified analysis was used to study the Ser326Cys polymorphism-bladder cancer relationship in general population, smoker and non-smokers, and people with different ethnic backgrounds.

## Materials and methods

### Publication search

We searched all published studies (prior to January 9, 2012) investigating the association between the *hOGG1* Ser326Cys polymorphism and bladder cancer risk in the PubMed database. A literature search was conducted using the search terms “human 8-oxoguanine DNA glycosylase,” “*hOGG1,” “OGG1,” “OGG,”* “polymorphism,” “genetic variation,” and “bladder cancer”. Review articles and reference cited in the searched studies were examined to identify additional published articles. The listed articles were assessed to determine whether they should be included in the meta-analysis. For studies with overlapping data published by same investigators, only the most recent or complete study was included. Conference abstracts, case reports, editorials, review articles, and letters were excluded. Studies included in the meta-analysis were required to meet the following criteria: 1) an unrelated case–control design was used, 2) genotype frequency was available, and 3) there is an evaluation of the *hOGG1* Ser326Cys polymorphism and bladder cancer risk.

### Data extraction

Two separate investigators reviewed and extracted data from all of the eligible publications independently, according to the inclusion and exclusion criteria listed above. The following information was extracted from each study: first author, year of publication, country of study population, genotyping method, genotype frequency, and the experimental design used to assess the effect of the *hOGG1* Ser326Cys polymorphism. Ethnic backgrounds were categorized as either Caucasian or Asian, and smoker status (smoker or non-smoker) was additionally recorded for the stratified analysis. Smokers included current smokers and former smokers. Non-smokers had never smoked.

### Statistical analysis

The meta-analysis evaluated the overall association between the *hOGG1* Ser326Cys polymorphism and the risk of bladder cancer using a number of methods. We evaluated the risk of the additive model (326Cys allele versus 326Ser allele), dominant model (Cys/Cys + Cys/Ser versus Ser/Ser), recessive model (Cys/Cys versus Cys/Ser + Ser/Ser) and the homozygote contrast (comparison of Cys/Cys versus Ser/Ser), respectively.

Hardy–Weinberg equilibrium (HWE) was tested using the Chi-squared test and it was considered statistically significant when *p* < 0.05. Sensitivity analysis was carried out by including and excluding studies not in HWE [24]. The heterogeneity of these studies was tested by the Q statistic [25] and was considered statistically significant when *p* < 0.10. The heterogeneity was quantified by the *I*^*2*^ metric, which is independent of the number of studies used in the meta-analysis (*I*^*2*^ < 25%, no heterogeneity; *I*^*2*^ = 25–50%, moderate heterogeneity; *I*^*2*^ > 50%, extreme heterogeneity) [26]. Publication bias was assessed by a funnel plot using both funnel plots and Egger’s linear regression test [27]. The combined odds ratio (OR) was estimated using fixed effects (FE) models with *p*_*heterogenity*_ ≥ 0.10, or random-effects (RE) models with *p*_*heterogenity*_ < 0.10 [28].

The overall association was measured by determining the OR with a corresponding 95% confidence interval (CI). The statistical significance of the overall OR was determined using a Z-test; *p* < 0.05 was considered statistically significant. Meta-analysis was performed using the Review Manager 5.0 software and Egger’s test was performed with STATA version 7.0.

## Results

### Study characteristics

After title and abstract screening, 10 case–control studies were found, however the studies by Kim et al.. [29] only demonstrated the effect of *hOGG1* Ser326Cys on muscle invasion of bladder cancer, and the studies by Wu et al. [30] and Huang et al. [21] , Gangwar et al. [18] and Mittal [31] contained overlapped data. The articles supplying more detailed information were brought into the analysis [21,31]. Thus, a total of 7 published studies met the inclusion criteria which included a case–control study design and published genotype frequencies. In all studies, the cases were histologically confirmed, and the controls were free of bladder cancer and matched for age and gender.

In total, 2474 bladder cancer cases and 2498 controls were included in the meta-analysis. We conducted subgroup analysis classified by ethnicity, hospital-based and HWE population. A summary of selected study characteristics is listed in Table 1, and genotype, allele frequencies and HWE information are shown in Table 2. Among all studies, four studies assessed Caucasian populations, including one from Spain [20], one from the USA [21] and two from Turkey [22,23]. Three studies were used to assessed Asian populations, including one from India [31], one from Korea [19] and one from Japan [17]. Of the seven final studies, six were hospital-based studies [19-23,31]. All studies but one [23] was consistent with HWE, and the total controls were not in HWE (*P* = 0.006).

**Table 1:** Characteristics of studies included in the meta-analysis

**First author [reference]**	**Country**	**Ethnicity**	**Genotyping method**	**Cases (age)**	**Controls (age)**	**Design of experiment**
Kim (2005) [19]	Korea	Asian	PCR-RFLP	N = 153 (62.9 ± 10.8 yrs)	N = 153 (60.7 ± 11.8 yrs)	Hospital-based
Karahalil (2006) [23]	Turkey	Caucasian	PCR-RFLP	N = 100 (mean age 60.2 yrs)	N = 100 (age, sex matched controls)	Hospital-based
Huang (2007) [21]	USA	Caucasian	Taqman	N = 696 (63.9 ± 11.1 yrs)	N = 629 (age, sex matched controls)	Hospital-based
Figueroa (2007) [20]	Spain	Caucasian	Taqman	N = 1150 (66 ± 10 yrs)	N = 1149 (65 ± 10 yrs)	Hospital-based
Arizono (2008) [17]	Japan	Asian	PCR-RFLP	N = 251 (68.2 ± 11.2 yrs)	N = 251 (age-matched healthy controls)	Population-based
Narter (2009) [22]	Turkey	Caucasian	PCR-RFLP	N = 83 (63.4 ± 11.7 yrs)	N = 45 (59.9 ± 9.71 yrs)	Hospital-based
Mittal (2011) [31]	India	Asian	ARMS-PCR	N = 212 (58.5 ± 12.4 yrs)	N = 250 (56.8 ± 10.8 yrs)	Hospital-based

**Table 2:** **Distribution of genotype and allele frequencies of the *hOGG1* Ser326Cys polymorphism**

**First author [reference]**	**Genotype**						**Allele**				**HWE( *p* )**
	**Cases *n* (%)**			**Controls *n* (%)**			**Cases *n* (%)**		**Controls *n* (%)**		
	**Ser/Ser**	**Ser/Cys**	**Cys/Cys**	**Ser/Ser**	**Ser/Cys**	**Cys/Cys**	**Ser**	**Cys**	**Ser**	**Cys**	
Kim (2005) [19]	37 (24.2%)	90 (58.8%)	26 (17.0%)	38 (24.8%)	70 (45.6%)	45 (29.4%)	164 (53.6%)	142 (46.4%)	146 (47.7%)	160 (52.3%)	0.30
Karahalil (2006) [23]	40 (40.4%)	47 (47.5%)	12 (12.1%)	62 (62.0%)	20 (20.0%)	18 (18.0%)	127 (64.1%)	71 (35.9%)	144 (72.0%)	56 (28.0%)	<0.001
Huang (2007) [21]	375 (61.2%)	209 (34.1%)	29 (4.73%)	348 (58.0%)	216 (36.0%)	36 (6%)	959 (78.2%)	267 (21.8%)	912 (76.0%)	288 (24.0%)	0.75
Figueroa (2007) [20]	649 (59.7%)	383 (35.2%)	56 (5.15%)	596 (58.5%)	361 (35.5%)	61 (5.99%)	1681 (77.3%)	495 (22.7%)	1553 (76.3%)	483 (23.7%)	0.52
Arizono (2008) [17]	61 (24.3%)	107 (42.6%)	83 (33.1%)	67 (26.7%)	135 (53.8%)	49 (19.5%)	229 (45.6%)	273 (54.4%)	269 (53.6%)	233 (46.4%)	0.20
Narter (2009)[22]	37 (63.8%)	13 (22.4%)	8 (13.8%)	18 (50.0%)	18 (50.0%)	0 (0%)	87 (75.0%)	29 (25.0%)	54 (75.0%)	18 (25.0%)	0.08
Mittal (2011) [31]	92 (43.4%)	93 (43.9%)	27 (12.7%)	122 (48.8%)	111 (44.4%)	17 (6.8%)	277 (65.3%)	147 (34.7%)	355 (71.0%)	145 (29.0%)	0.21
Total	1291 (52.2%)	942 (38.1%)	241 (9.74%)	1251 (52.0%)	931 (38.7%)	226 (9.39%)	3524 (71.2%)	1424 (28.8%)	3433 (71.3%)	1383 (28.7%)	0.006

Four of the seven eligible studies provided data on smoker and non-smoker subjects [17,21,23,31]. Distribution of genotype and allele in the smoker versus non-smoker groups is shown in Table 3. However, one of this four studies only showed the data in dominant model [31], while another did in recessive model [21]. These studies were analyzed according to smoker status in different models.

**Table 3:** ** *hOGG1* Ser326Cys genotype frequency and distribution according to smoking status**

	**First author [reference]**	**Genotype**					
		**Cases *n* (%)**			**Controls *n* (%)**		
		**Ser/Ser**	**Ser/Cys**	**Cys/Cys**	**Ser/Ser**	**Ser/Cys**	**Cys/Cys**
**Smoker**	Karahalil (2006)[23]	14 (41.2%)	16 (47.1%)	4 (11.7%)	27 (62.8%)	9 (20.9%)	7 (16.3%)
	Huang (2007) [21]	279 (62.7%)	166 ^a^ (37.3%)	183 (55.5%)		147^a^ (44.5%)	
	Arizono (2008) [17]	42 (25.5%)	72 (43.6%)	51 (30.9%)	42 (26.6%)	83 (52.5%)	33 (20.9%)
	Mittal (2011) [31]	107 ^b^ (89.2%)	13 (10.8%)	72 ^b^ (93.5%)		5 (6.49%)	
**Non-smoker**	Karahalil (2006) [23]	7 (53.8%)	4 (30.8%)	2 (15.4%)	38 (66.7%)	9 (15.8%)	10 (17.5%)
	Huang (2007) [21]	96 (60.8%)	62 ^b^ (39.2%)	165 (59.4%)		113 ^b^ (40.6%)	
	Arizono (2008) [17]	19 (22.1%)	35 (40.7%)	32 (37.2%)	25 (26.9%)	52 (55.9%)	16 (17.2%)
	Mittal (2011) [31]	75 ^a^ (84.3%)	14 (15.7%)	161 ^a^ (93.1%)		12 (6.94%)	

a: As only the data of dominant model were available, these data were referred to the sum of Ser/Cys and Cys/Cys.

b: As only the data of recessive model were available, these data were referred to the sum of Ser/ Ser and Ser/Cys.

### Main meta-analysis results

**Figure 1:**
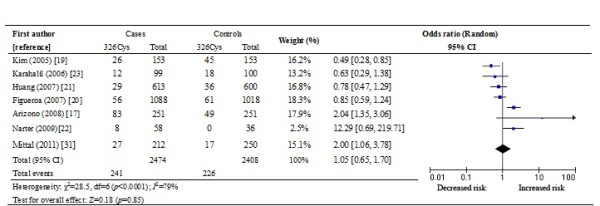
Overall meta-analysis of the 326Cys allele in the recessive genetic model using a random-effect model.

The heterogeneity results and the determined association between the *hOGG1* 326Cys polymorphism and bladder cancer risk are shown in Table 4. Overall results showed that individuals carrying the *hOGG1* 326Cys allele in the additive model did not have significantly increased risk for bladder cancer compared to those carrying the 326Ser allele (OR: 1.06, 95% CI: 0.96-1.26; *p* = 0.49) in the additive model. Similarly, no significant difference in bladder cancer risk was found between the patients with a Cys/Cys genotype and those with a Ser/Ser genotype (OR 1.11, 95% CI = 0.74-1.66, *p* = 0.63) in homozygote contrast. This was also the case for Cys/Cys versus Ser/Cys + Ser/Ser in the recessive genetic model (OR: 1.05, 95% CI: 0.65-1.70; *p* = 0.85) and for Cys/Cys + Ser/Cys versus Ser/Ser in the dominant genetic model (OR: 1.07, 95% CI: 0.87-1.32; *p* = 0.53) (Table 4 and Figure 1).

**Table 4:** **The main ORs of *hOGG1* Ser326Cys polymorphisms in the meta-analysis**

**Allele and genotype**	**Populations**	**OR**	** *I* **^** *2* **^**(%)**	** *P* **_**heterogeneity**_	**Analysis model**	** *P* **
326Cys allele versus 326Ser allele (additive model) ^a^	All populations	1.06 (0.90, 1.26)	64%	0.01	Random	0.49
	Caucasian populations	0.95 (0.85, 1.06)	30%	0.23	Fixed	0.38
	Asian populations	1.14 (0.82, 1.57)	75%	0.02	Random	0.44
	Hospital-based	1.00 (0.90, 1.11)	51%	0.07	Random	0.99
	Studies in HWE	1.01 (0.91, 1.44)	65%	0.02	Random	0.83
Cys/Cys versus Ser/Ser (homozygote contrast) ^a^	All populations	1.11 (0.74, 1.66)	65%	0.009	Random	0.63
	Caucasian populations	0.87 (0.66, 1.16)	0%	0.41	Fixed	0.35
	Asian populations	1.34 (0.64, 2.83)	78%	0.01	Random	0.44
	Hospital-based	0.97 (0.65, 1.45)	54%	0.06	Random	0.89
	Studies in HWE	1.12 (0.71, 1.79)	71%	0.004	Random	0.62
Cys/Cys versus Cys/Ser + Ser/Ser (recessive genetic model)^a^	All populations	1.05 (0.65, 1.70)	79%	<0.0001	Random	0.85
	Caucasian populations	0.85 (0.65, 1.21)	25%	0.26	Fixed	0.25
	Asian populations	1.26 (0.50, 3.17)	89%	<0.0001	Random	0.62
	Hospital-based	0.89 (0.57, 1.38)	66%	0.01	Random	0.59
	Studies in HWE	1.14 (0.67, 1.94)	81%	<0.0001	Random	0.64
Cys/Cys + Cys/Ser versus Ser/Ser (dominant genetic model) ^a^	All populations	1.07 (0.87, 1.32)	58%	0.03	Random	0.53
	Caucasian populations	1.04 (0.74, 1.47)	75%	0.007	Random	0.82
	Asian populations	1.16 (0.91, 1.47)	0%	0.85	Fixed	0.23
	Hospital-based	1.07 (0.83, 1.36)	64%	0.02	Random	0.61
	Studies in HWE	0.97 (0.86, 1.09)	0%	0.45	Fixed	0.59

a: The reference groups are the second genotypes.

These seven studies were analyzed by stratified analysis. However, no increased risk was detected in Asians or Caucasians either in the additive model, the homozygote contrast, the recessive model, or the dominant model (all *p* > 0.05). Furthermore, no statistically significant conclusions were found for hospital-based subjects in the various statistical models (all *p* > 0.05). When meta-analysis of studies in HWE was conducted, no significant correlation was detected in any type of statistical model, either (all *p* > 0.05).

### Meta-analysis with different smoking status

In the stratified analysis of the effect of smoker status, there were four studies discussing the interaction of smoking behavior and *hOGG1* Ser326Cys (Table 5). Due to the missing data, three studies were included in dominant or recessive model analysis, while two studies were in additive model or homozygote contrast. Overall, there were 1372 smokers (764 case and 608 controls) and 947 non-smokers (346 cases and 601 controls) included in this part of meta-analysis. Two studies assessed Asian population [17,31] and the other two assessed Caucasian populations [21,23]. The samples in this analysis with different smoking status were small, and then further stratified analysis was not performed in this part of meta-analysis.

**Table 5:** **The ORs of *hOGG1* Ser326Cys polymorphisms according smoker status**

**Allele and genotype**	**Populations**	**OR**	** *I* **^** *2* **^**(%)**	** *P* **_**heterogeneity**_	**Analysis model**	** *P* **
326Cys allele versus 326Ser allele (additive model) ^a^	Smoker subjects	1.29 (0.97-1.71)	0%	0.64	Fixed	0.08
	Non-smoker subjects	**1.59 (1.08, 2.32)**	0%	0.65	Fixed	0.02
Cys/Cys versus Ser/Ser (homozygote contrast) ^a^	Smoker subjects	1.46 (0.84, 2.56)	0%	0.66	Fixed	0.18
	**Non-smoker subjects**	**2.93 (1.43, 5.99)**	**37%**	**0.21**	**Fixed**	**0.003**
Cys/Cys versus Cys/Ser + Ser/Ser (recessive genetic model) ^a^	Smoker subjects	1.54 (1.00, 2.37)	0%	0.44	Fixed	0.05
	**Non-smoker subjects**	**2.41 (1.47, 3.95)**	**0%**	**0.42**	**Fixed**	**0.0005**
Cys/Cys + Cys/Ser versus Ser/Ser (dominant genetic model) ^a^	Smoker subjects	0.72 (0.26, 2.02)	87%	0.004	Random	0.53
	Non-smoker subjects	1.58 (0.72, 3.50)	75%	0.02	Random	0.26

^a:^ The reference groups are the second genotypes.

**Figure 2:**
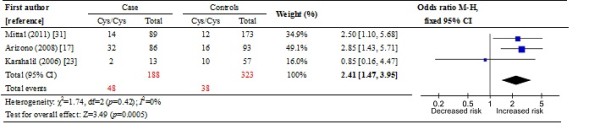
Overall meta-analysis of the Cys/Cys genotype in the recessive genetic model in the non-smoker population.

In the non-smoker population, a significant association was found between the *hOGG1* 326Cys allele and bladder cancer risk in the recessive model (OR: 2.41, 95% CI: 1.47-3.95; *p* = 0.0005). Similarly, this association was found in the homozygote contrast (OR: 2.93, 95% CI: 1.43-5.99; *p* = 0.003) and the additive model (OR: 1.59, 95% CI: 1.08-2.32; *p* = 0.02) (Tables 5 and Figure 2). However, no significant association was found in the smoker population, between the *hOGG1* 326Cys allele and bladder cancer risk (all *p* > 0.05). This finding suggests a dominant effect of *hOGG1* 326Cys on bladder cancer risk among the non-smokers.

### Test of heterogeneity, sensitivity analysis and publication bias

Significant heterogeneity between studies was observed in some comparisons, and the detailed data were shown in Table 4. An extreme heterogeneity between these studies was detected in the first meta-analysis (*I*^*2*^ = 64%, *P*_heterogeneity_ = 0.02). Then, the random-effects (RE) models were used to evaluate the combined ORs when necessary. Sensitivity analysis was performed both by sequential removal (statistics of study removal) of individual studies and cumulative statistics on the comparisons of worldwide subjects. The combined ORs of the *hOGG1* Ser326Cys polymorphism were not influenced by either the addition or removal of any individual study, such as the study from Narter et al. [22] with the genotype distribution in the control group deviating from HWE.

Funnel plots were performed to assess the publication bias in this meta-analysis and showed a symmetrical inverse funnel shape (data not shown). The funnel results suggested that the selection of publications was an unlikely source of bias in the meta-analysis of the association between the *hOGG1* Ser326Cys polymorphism and bladder cancer.

## Discussion

DNA damage generated by different carcinogenic agents can be repaired primarily through base excision repair (BER) pathway, composed of many DNA repair genes. Common polymorphisms in DNA repair genes may alter protein function and the possibility to repair damaged DNA. Defects in DNA repair pathways may lead to genetic instability and carcinogenesis [32,33]. The *hOGG1* gene is a key gene in the BER pathway and DNA repair process, and the Ser326Cys polymorphism is reported to be a functional variation in the *hOGG1* gene.

Since the original identification of the *hOGG1* Ser326Cys polymorphism, a number of studies have investigated the genetic effect of this polymorphism on bladder cancer susceptibility. In the eligible studies, the percentage of 326Cys allele was 0.523 in the Korean population (n = 153), 0.464 in the Japanese population (n = 251), and 0.290 in the Indian population (n = 250), while it was 0.280, 0.240, 0.237 and 0.250, respectively, for the Caucasian population in Turkey (n = 100), USA (n = 600), Spain (n = 1018) and Turkey (n = 36). Considering these inconsistent findings, we performed a meta-analysis on these studies to quantify the available data and generate a robust estimate of the effect of the Ser326Cys polymorphism on bladder cancer. Meta-analysis has been proved to be a powerful method from a relatively large number of subjects [34,35].

In this study, we analyzed the data from seven available case–control studies. The results are conflicting about the role of the *hOGG1* Ser326Cys polymorphism in relation to bladder cancer susceptibility. Three studies found an increased risk for bladder cancer associated with the 326Cys allele [17,23,31], two studies identified a reduced risk [19,21], and the other two did not detect the association between Ser326Cys polymorphism and bladder cancer [20,22].

When all the eligible studies were pooled into analysis, it failed to uncover any evidence that there was an association between the Ser326Cys polymorphism and bladder cancer susceptibility overall. No statistical evidence was found in a recessive model, either in a dominant model, an additive model or a homozygote contrast. Moreover, the association of the Ser326Cys polymorphism and bladder cancer could not be found in Asians or Caucasians using various models. Either in the hospital-based subpopulation or the population in HWE, no significant conclusion were found. Taken together, it may be concluded that *hOGG1* Ser326Cys polymorphism lacks association with bladder cancer risk in such common population.

Cigarette smoking is a major risk factor for bladder cancer, and the association between smoking status and bladder cancer risk remains a point of controversy. Some studies have found that cigarette smoking was more strongly associated with increased risk of invasive bladder cancer than with low-grade superficial bladder cancer [36]. Even among lifelong non-smoker populations, environmental tobacco smoke (ETS) exposure can still be a risk factor for bladder cancer in women [24]. Therefore, meta-analysis was further performed among smokers and non-smokers, to clarify the effect of smoking behavior on this relationship. Interestingly, in non-smokers, the analysis for combined data suggested a remarked association between the *hOGG1* Ser326Cys polymorphism and bladder cancer risk. This association was confirmed in the additive model, homozygote contrast and recessive genetic model, with the OR values of 1.59 (*p* = 0.02), 2.93 (*p* = 0.003) and 2.41 (*p* = 0.005), respectively. However, when the parallel analysis was performed in smokers, no significantly statistical conclusions were found in all genetic models (all *p* > 0.05).

It is well established that the carcinogenesis of bladder cancer is a result of the interaction between environmental factors and genetic background. Besides the role of genetic variants, smoking behavior shows a major effect on the bladder cancer susceptibility [4]. Smoking status contributed to the heterogeneity in *hOGG1* Ser326Cys estimates, since the frequencies of variant alleles altered with different smoking behaviors [17,21,23,31]. Thus, the association of *hOGG1* Ser326Cys polymorphism and bladder cancer risk might alter under different smoking status. Actually, when the eligible studies were pooled into meta-analysis, a significant risk was shown in the non-smoker subgroup, but not in the smoker subgroup. Therefore, the genetic effect may be more dominant in those who have not been exposed to an environmental risk factor. Notably, the results should be interpreted with caution, since only limited studies are included in this part of analysis.

The limitations of the present study include that 6 out of the 7 studies in this meta-analysis are hospital-based and only 1 is population-based, and the heterogeneity and publication bias may exist due to the limited sample size, and the variation in the genotyping methods and experimental designs. Moreover, the real function of the Ser326Cys polymorphism may have an effect only under special conditions of cellular oxidative stress or tumor types [37]. Among the common complicated population, we could not detect significant association between *hOGG1* Ser326Cys and bladder cancer risk among overall studies, and Asian, Caucasian and hospital-based subpopulation. In addition, one study from Karahalil et al. [23] was not in HWE among the controls. But, the OR was not substantially altered when the subpopulation in HWE was pooled. It might due to that the weight of this study was only 5 % and is unlikely to influence much of our results. Moreover, the combined ORs of the *hOGG1* Ser326Cys polymorphism were not influenced by any individual study. All of these suggest that well-designed and prospective studies with larger sample sizes should be conducted to clarify the role of *hOGG1* Ser326Cys polymorphism in bladder carcinogenesis.

## Conclusions

In summary, our meta-analysis suggests the *Ser326Cys* polymorphism lacks association with bladder cancer risk in common population, but specifically increases the susceptibility for non-smoker populations. Further studies are needed, especially to investigate the effect of the gene-environment interaction.

## Competing interests

The authors declare that there are no competing interests.

## Authors’ contributions

CJ participated in collection of data and manuscript preparation. ZL and HC performed the statistical analysis. CJ and HC participated in study design and critically revised the manuscript. CL and HG participated in study design and manuscript preparation. All authors read and approved the final manuscript.

## Pre-publication history

The pre-publication history for this paper can be accessed here:

http://www.biomedcentral.com/1471-2407/12/335/prepub
